# The In Vitro Enzymatic Degradation of Cross-Linked Poly(trimethylene carbonate) Networks

**DOI:** 10.3390/polym9110605

**Published:** 2017-11-13

**Authors:** Zhipeng Hou, Jianshe Hu, Jianxin Li, Wei Zhang, Miao Li, Jing Guo, Liqun Yang, Zhangpei Chen

**Affiliations:** 1 Center for Molecular Science and Engineering, College of Science, Northeastern University, Shenyang 110819, China; zhp.hou@gmail.com (Z.H.); chenzhangpei@mail.neu.edu.cn (Z.C.); 2 Key Laboratory of Reproductive Health and Medical Genetics, National Health and Family Planning Commission, Shenyang 110031, China; jxinl@vip.sina.com (J.L.); zhangwei@lnszjk.com.cn (W.Z.); limiao_lnszjk@163.com (M.L.); 15804003187@163.com (J.G.); 3 Key Laboratory of Reproductive Health, Liaoning Research Institute of Family Planning, Shenyang 110031, China

**Keywords:** cross-linked poly(trimethylene carbonate) networks, enzymatic degradation, initial molecular weight, cross-linker amount, form-stability

## Abstract

The in vitro enzymatic degradation of cross-linked poly(trimethylene carbonate) networks (PTMC-Ns) was performed in lipase solutions at 37 °C, and the effect of the initial molecular weight and cross-linker amount as well as the cross-linker type on the degradation rate of PTMC-Ns was investigated. Due to their denser structure and more hydrophobic surface as well as the higher glass transition temperature, a slower degradation rate was seen for PTMC-Ns with high initial molecular weight at a given cross-linker amount. Similar results could be observed as the cross-linker amount increased, and cross-linker type also influenced the degradation rate of PTMC-Ns. Furthermore, the enzymatic degradation of PTMC-Ns was accelerated by the surfactants role of lipase via surface erosion mechanism, the enzymatic degradation rate was higher than that of hydrolysis case. The results indicated that PTMC-Ns were promising candidates for clinical subcutaneous implants, especially due to their tunable degradation rate and enhanced form-stability as well as no acidic degradation products.

## 1. Introduction

As one of the most important biodegradable polymers, poly(trimethylene carbonate) (PTMC) has attracted considerable attention in recent years due to its favorable characteristics, such as well-documented biocompatibility, excellent biodegradability and rubber-like properties [1,2,3]. On top of that, the degradation products of PTMC are not acidic, which is much better than polyesters to avoid inflammation [4,5,6,7] led by acidic degradation products. Therefore, PTMC has been one of the promising candidate materials for biomedical and pharmaceutical applications including drug delivery systems [8,9,10,11,12], nerve guides [13,14,15] and temporary three-dimensional (3D) scaffolds in tissue engineering [16,17,18,19,20]. However, further study showed poor compatibility between degradation rate and form-stability of PTMC during in vivo degradation [21]; for example, although the degradation rate was slow, the form-stability for low-molecular-weight PTMC was poor; while the form-stability was enhanced, high-molecular-weight PTMC had an undesired faster degradation rate in vivo. It would greatly hinder the extensive application of PTMC in biomedical fields. The exact combination of good form-stability and low degradation rate of PTMC needs to be achieved urgently.

Cross-linking normally gives polymers stable dimensions and enhanced corrosion resistance [22,23], which is considered as one of effective strategies to combine good form-stability and low degradation rate for PTMC. In our previous works, the cross-linked PTMC networks (PTMC-Ns) were fabricated using a new reaction methodology of cross-linking agent [24,25] rather than gamma irradiation to avoid the chain scission [26,27]. The results showed that the mechanical and thermal properties of the structurally stable PTMC-Ns were preferable than that of PTMC [24]. Moreover, the PTMC-Ns properties could be predictably controlled and tailed by varying the polymer composition and cross-linker amount [25]. While the synthesis and properties have been studied extensively, reports on the degradation in vitro and in vivo of PTMC-Ns are limited. Bat et al. [27,28,29] investigated the in vitro and in vivo degradation of the PTMC-Ns based on the gamma irradiated (co)polymer films of trimethylene carbonate (TMC) and ε-caprolactone (CL). The results of the in vitro enzymatic degradation revealed that the degradation of networks was in accord with a surface erosion mechanism and the erosion rates could be tuned linearly from 0.8 to 45 mg/(cm^2^ × day) by varying the TMC to CL ratio and the irradiation dose [27]. The Gamma irradiated networks also eroded upon implantation in the subcutaneous tissue of rats. The results showed that increasing the irradiation dose led to lower erosion rates and to a milder tissue response [28,29]. In our previous works, PTMC-Ns exhibited markedly slower degradation rate and better form-stability than that of the un-crosslinked samples [25], and presented a surface erosion mechanism upon in vivo degradation [30]. Both results indicated that resorbable and form-stable PTMC-Ns with tunable properties may find application in a broad range of biomedical applications.

Lipase (from *Thermomyces lanuginosus*, ≥100,000 U/g) is a good model for the in vivo erosion of PTMC-based materials [2,21,31], and it also plays an important role in degradation of PTMC-Ns [25,30]. In this paper, the in vitro enzymatic degradation of the PTMC-Ns performed in lipase solutions (from *Thermomyces lanuginosus*, ≥100,000 U/g) at 37 °C, was studied in more details. The effect of initial molecular weight and cross-linking density as well as the cross-linker type on degradation of PTMC-Ns was investigated, and the degradation rate and form-stability were monitored during the enzymatic degradation process. The hydrolytic degradation was also performed in pH = 7.4 phosphate buffered saline (PBS) to elucidate the role of enzymes in the degradation of PTMC-Ns.

## 2. Materials and Methods

### 2.1. Materials

TMC was purchased from Daigang Biomaterial Co., Ltd (Jinan, China), recrystallized twice from acetic ether and dried for 24 h at 37 °C under reduced pressure before polymerization. Stannous octoate [Sn(Oct)_2_] (95%) and lipase solutions (from *Thermomyces lanuginosus*, ≥100,000 U/g) were used as received from Sigma-Aldrich. All other solvents and reagents used were of analytical grades and purified by standard methods.

### 2.2. Measurements

The initial molecular weight (Mn) was determined by GPC (Waters, Milford, MA, USA) with a Waters Model 1515 isocratic high-performance liquid chromatography pump, a Waters Model 2414 differential refractive index detector and a Waters Styragel HT4 chromatographic column using THF as the solvent and a flow rate of 1 mL/min at 35 °C. The water contact angles of the different PTMC-Ns were determined using a contact angle-measuring instrument (Kruss, Hamburg, Germany) equipped with a video measuring system was used to determine static contact angles of sessile drops (each based on 6 different films) at room temperature. The phase transition temperature of PTMC-Ns was determined with a Netzsch DSC 200 F3 (Netzsch, Serb, Germany) equipped with a liquid nitrogen cooling system. The measured temperature range was between −100 and 100 °C, and the heating rate was 10 °C/min under nitrogen atmosphere. The thermal stability of the polymers under nitrogen atmosphere used a Netzsch TGA 209 F3 (Netzsch, Serb, Germany) from room temperature to 550 °C and a heating rate of 10 °C/min. The mechanical properties of the networks were measured using an Instron 1121 universal testing machine (Instron, Boston, MA, USA) with a crosshead speed of 50 mm/min in accordance with GB/T 1040.1-2006. The tests were done on triplicate samples, and the results were presented as an average of the tested samples. The scanning electron microscopy (SEM) images were obtained with a XL30ESEM–FEG microscope (FEI-Philips, Amsterdam, The Netherlands). The sample surfaces were coated with Au to avoid charging. The pH of the media before and after degradation was monitored with a combination microelectrode (InLabMicro™, Mettler-Toledo, Zurich, Switzerland,) and a digital pH meter (FiveEasy™, Mettler-Toledo, Zurich, Switzerland).

### 2.3. Fabrication of PTMC-Ns Rods

The PTMC-Ns rods were directly synthesized via bulk ring-opening polymerization reaction of TMC with cross-linker bis(trimethylene carbonate) (BTB) or bis(ε-caprolactone) (BCP) in the presence of Sn(Oct)_2_ as the catalyst. Under nitrogen atmosphere, dried TMC and cross-linker with anhydrous toluene solution of Sn(Oct)_2_ were added to glass ampoules containing clean and dry Teflon tubes with an inner diameter of 3 mm; The toluene was removed afterwards by evacuation. The ampoules were purged with dry nitrogen, heat-sealed under vacuum and vigorously shaken to obtain a homogeneous mixture of the monomers and the catalyst. Then, the ampoules were conditioned in an oil bath pre-heated to 130 ± 2 °C for 24 h. After the reaction, the ampoules were quenched at room temperature, and the PTMC-Ns rods were easily removed from the Teflon tubes and used without any further purification.

To determine the gel percentage and swelling degree of the resulting PTMC-Ns, a piece of network sample was weighed (*w*_0_) and kept in a sealed flask containing chloroform. At regular intervals, the sample was removed and the excess solvent was blotted. The sample was then weighed and returned to the medium. This procedure was continued until a constant weight was attained (*w*). The gel percentages were determined gravimetrically after the swollen gels were dried to constant weights (*w*_1_). The gel percentage and swelling degree were calculated according to Equations (1) and (2), respectively.

(1) $$\mathrm{Gel}\;\mathrm{percentage}(\%)=\frac{w_{1}}{w_{0}}\times 100$$

(2) $$\mathrm{Swelling}\;\mathrm{degree}(\%)=\frac{w-w_{1}}{w_{1}}\times 100$$

where *w*_0_ is the initial weight of the samples before swelling, *w*_1_ is the final weight of the dried (extracted) samples, and *w* is the weight of the swollen samples. These measurements for gel percentage and swelling degree were done in triplicate for each network sample.

### 2.4. In Vitro Enzymatic Degradation

According to previous reports [2,21,31], lipase can effectively degrade PTMC. PTMC-Ns rods with a length of 10 mm were weighed and conditioned in lipase solutions (from *Thermomyces lanuginosus*, ≥100,000 U/g), which were refreshed twice a week. The degradation experiments were performed in triplicate at 37 °C with gentle shaking. At regular time intervals, the PTMC-Ns rods were removed from the degradation media and washed with distilled water. After blotted with a tissue, the specimens were weighted and then vacuum dried at 37 °C to a constant weight for further analysis, and the diameter of the dried specimens was recorded. The pH of the media containing degradation products was also measured. The mass loss and loss in diameter were calculated via the following equations:

(3) $$\mathrm{Mass}\;\mathrm{loss}(\%)=\frac{w_{i}-w_{d}}{w_{i}}\times 100$$

(4) $$\mathrm{Water}\;\mathrm{uptake}(\%)=\frac{w_{w}-w_{d}}{w_{d}}\times 100$$

(5) $$\mathrm{Loss}\;\mathrm{in}\;\mathrm{diameter}(\%)=\frac{d_{i}-d_{d}}{d_{i}}\times 100$$

where *w_i_*, *w_w_* and *w_d_* represent the initial weight, wet weight and dry weight of the samples, respectively; and *d_i_* and *d_d_* represent the initial diameter and final diameter of the dry samples after degradation, respectively.

As a control, the hydrolytic degradation of the PTMC-Ns rods was carried out similarly with the enzymatic degradation, except that the medium was pH = 7.4 PBS solutions in absence of lipase.

## 3. Results and Discussion

### 3.1. Synthesis and Properties of PTMC-Ns

To investigate the effect of initial molecular weight and cross-linking amount as well as the cross-linker type on degradation, PTMC-Ns were fabricated in different cross-linking conditions, as described in our previous works [25]. Briefly, linear PTMC with four different molecular weights in the range of 72–329 kg/mol was cross-linked by 0.1 mol % BTB to prepare PTMC-Ns, and the molecular weight of the linear PTMC was regarded as the initial molecular weight of the PTMC-Ns. Besides, PTMC-Ns were obtained via the cross-linking of PTMC (Mn = 256 kg/mol) with varied BTB amount from 0 to 0.5 mol %. To investigate the effect of cross-linker type, PTMC (Mn = 256 kg/mol) was cross-linked by 0.1 mol % BTB and BCP, respectively. The properties of the obtained PTMC-Ns are shown in Table 1.

As shown in Table 1, the cross-linking conditions greatly influenced the properties of the resulting PTMC-Ns. By increasing the initial molecular weight and by increasing the cross-linker amount, higher gel percentage and lower swelling degree as well as enhanced thermal and mechanical properties were recorded for the obtained PTMC-Ns. For example, at a given initial molecular weight of 256 kg/mol, the gel percentage increased from 80.94 ± 3.23% to 97.89 ± 1.1% and the swelling degree decreased from 3943 ± 103% to 498 ± 16% as the BTB amount increased from 0.05 to 0.5 mol %, while, at the given BTB amount of 0.1 mol %, the gel percentage increased from 39.85 ± 1.98% to 97.96 ± 1.29% and the swelling degree decreased from 9916 ± 270% to 567 ± 25% with the increase of initial molecular weight from 72 to 329 kg/mol. Similar tendency could be observed for the thermal and the mechanical properties of the PTMC-Ns. Table 1 also showed that the PTMC-Ns cross-linked by BTB had slightly stronger physical properties than that of counterparts cross-linked by BCP. It was because of the more similar structure and reactivity of BTB than that of BCP to TMC monomer.

### 3.2. Mass Loss and Degradation Rate

**Figure 1:**
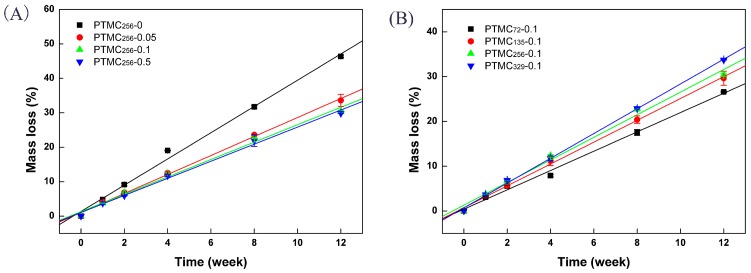
The mass loss of bis(trimethylene carbonate) (BTB) cross-linked PTMC-Ns with: different cross-linker amount (**A**); and different initial molecular weight (**B**), during in vitro enzymatic degradation as a function of time.

It has been reported that lipase is one of the most efficient enzymes in eroding PTMC polymers [32]. The enzymatic degradation of the resulting PTMC-Ns was performed in vitro in the presence of the lipase (from *Thermomyces lanuginosus*, ≥100,000 U/g) at 37 °C. Figure 1 shows the profiles of the mass loss of the PTMC-Ns as a function of the degradation time.

It was clearly seen that the mass loss of the PTMC-Ns was dependent on the cross-linker amount and the initial molecular weight. Figure 1A showed that the mass loss of PTMC-Ns cross-linked by higher cross-linker amount was lower, whereas the networks cross-linked by lower cross-linker amount lost their mass in a much faster way. The mass loss of the PTMC_256_-0, PTMC_256_-0.05, PTMC_256_-0.1 and PTMC_256_-0.5 was 46.3 ± 0.4%, 33.6 ± 1.8%, 30.4 ± 0.4% and 29.8 ± 0.03%, respectively, after 12 weeks. Furthermore, the PTMC initial molecular weight also had conspicuous effect on the enzymatic degradation behavior of the PTMC-Ns. As shown in Figure 1B, the mass loss of PTMC-Ns degraded in lipase increased with the initial molecular weight. For instance, at a given BTB amount of 0.1 mol %, the PTMC_72_-0.1 and PTMC_135_-0.1 displayed a mass loss of 26.6 ± 0.2% and 29.6 ± 1.6%, respectively, after 12 weeks, whereas the mass loss for PTMC_329_-0.1 was 33.7 ± 0.05% at the same time point.

To quantitatively elucidate the degradation behavior, the degradation rate constant of the PTMC-Ns was calculated from the mass loss based on a first-order kinetic model, and the following equation was adopted:

(6) $$M_{t2}=M_{n}-k(t_{2}-t_{1})$$

where *k* is the degradation rate constant and *M_t_* is mass loss of PTMC-Ns at the degradation time of *t*.

The results determined from linear fitting of the data in the plots of mass loss versus the degradation time (Figure 1) are listed in Table 2 and Table 3. Apparently, the degradation rate of the PTMC-Ns was lower versus that of PTMC and decreased with increasing the cross-linker amount, as shown in Table 2. The degradation rate of the non-crosslinked PTMC_256_-0 was 3.81 %/w, which was higher than 2.76 %/w of the 0.05 mol % BTB cross-linked PTMC network (PTMC_256_-0.05). Then, the degradation rate of the PTMC_256_-Ns further decreased to 2.48 %/w as the cross-linker amount reached to 0.5 mol %. The results indicate that cross-linking could result in networks with much reduced degradation rate [33], as the cross-linker amount increased from 0 to 0.5 mol %. It was due to that gel with three-dimensional structures was insensitive to the enzymes attack, the PTMC-Ns with higher gel percentage have higher anti-degradation ability. The gel percentage for the PTMC_256_-Ns cross-linker by 0.05–0.5 mol % BTB was in a range of 80.94 ± 3.23%–97.89 ± 1.1%, indicating the formation of much denser networks that make the carbonate bonds less accessible for enzymatic hydrolysis.

**Figure 2:**
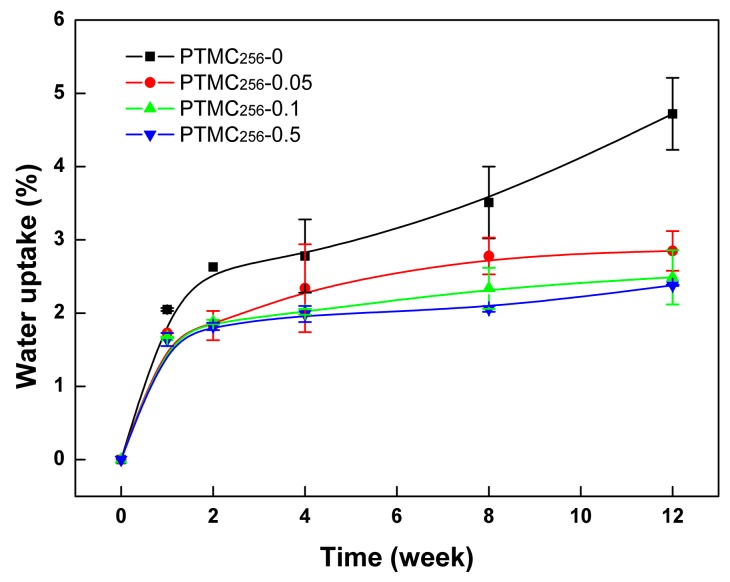
The water uptake of BTB cross-linked PTMC_256_-Ns with different cross-linker amount during in vitro enzymatic degradation as a function of time.

It is well known that the mobility of the polymer chain is an important factor that influences the polymers degradation [34]. The dense network formation could weaken the chain mobility of PTMC-Ns, it would make the chains more inaccessible to the active site of the enzymes, and then reduce the degradation rate. On the other hand, water uptake occurred during the degradation and appeared to be an important factor in the enzymatic degradation of PTMC-Ns. The permeated water molecules could lead to plasticization of the networks, which would enhance the polymer chain mobility and thus facilitate enzymatic attack. As mentioned earlier, the swelling degree of PTMC_256_-Ns in chloroform decreased from 3943 ± 103% to 498 ± 16% with the increase of BTB amount from 0.05 to 0.5 mol %. This indicated that the formed networks were densely cross-linked, almost no space so that water could easily penetrate into the networks. Thus, the water uptake of the PTMC_256_-Ns decreased with the increase of BTB amount (Figure 2). As a result, we concluded that a higher cross-linking density in the PTMC_256_-Ns cross-linked by higher BTB amount enabled it to be degraded slower.

The in vitro enzymatic degradation rate of the PTMC-Ns seemed to be initial molecular weight dependent. As shown in Table 3, at a given BTB amount of 0.1 mol %, the degradation rate of the PTMC-Ns with low initial molecular weight of 72 kg/mol (PTMC_72_-0.1) was 2.16 %/w, and then it was increased to 2.44 %/w for PTMC_135_-0.1 as the initial molecular weight reached to 135 kg/mol. As the initial molecular weight further increased to 256 and 329 kg/mol, the degradation rate of PTMC_256_-0.1 and PTMC_329_-0.1 was 2.52 %/w and 2.78 %/w, respectively; obviously, the higher the initial molecular weight, the greater the degradation rate of the PTMC-Ns.

**Figure 3:**
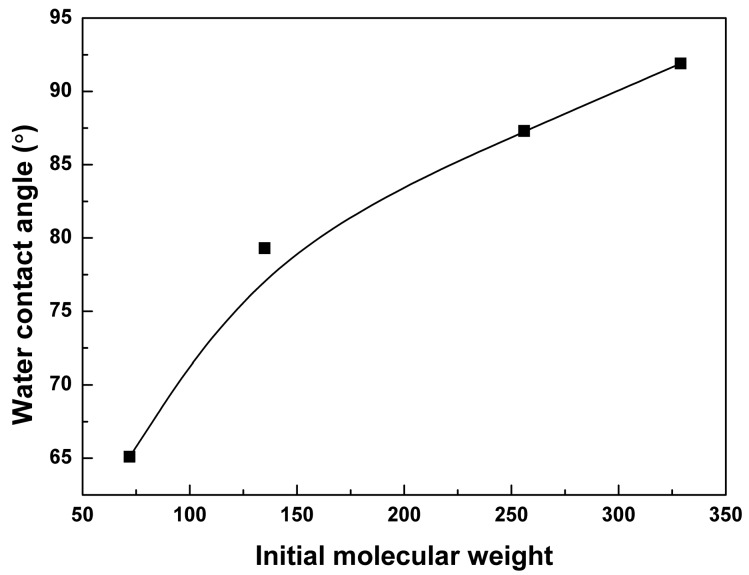
The water contact angle of 0.1 mol % BTB cross-linked PTMC-Ns was a function of the initial molecular weight.

This phenomenon was related to the hydrophilicity, which has an important effect on the polymer degradation behavior [35]. As shown in Figure 3, the static contact angle of the 0.1 mol % BTB cross-linked PTMC-Ns increased with the increase of the initial molecular weight, reaching a maximum of 91.9° at the 326 kg/mol. The results illustrated that PTMC samples became more hydrophobic as the initial molecular weight increased. It has been reported that the lipase activity is exhibited much higher on the hydrophobic surface [36,37]. Hence, the lipase activity is high in degrading the PTMC-Ns with high initial molecular weight, owing to their hydrophobic surfaces. The result was in coincidence with that of the in vitro enzymatic degradation of the pure linear PTMC with different initial molecular weight in our previous works [30].

**Figure 4:**
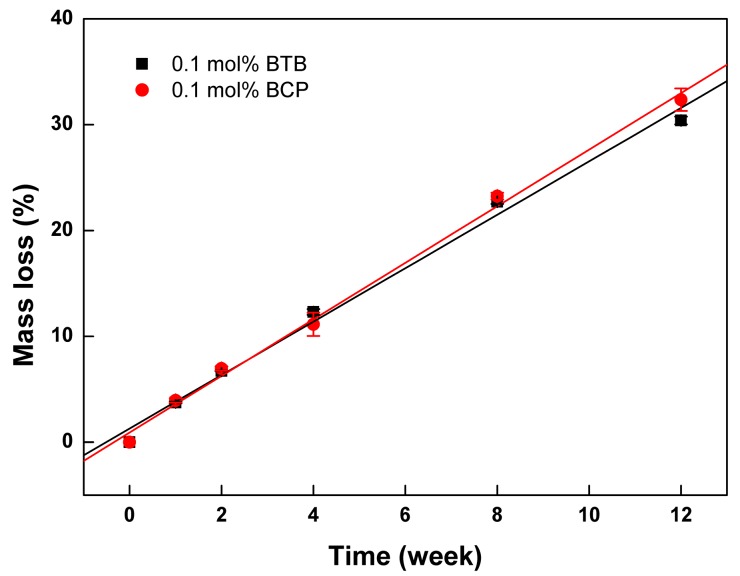
The mass loss of BTB and BCP cross-linked PTMC_256_-0.1 during in vitro enzymatic degradation as a function of time.

The effect of the cross-linker type on the degradation behavior of the resulting PTMC-Ns was also investigated, and the results were displayed in Figure 4. It was found that the mass loss of PTMC_256_-0.1 cross-linked by BTB or BCP increased gradually with degradation. The linear relationships between the mass loss and degradation time were determined for the both cases, and the degradation rate calculated based on Equation (4) was 2.52 %/w (R = 0.996) and 2.67 %/w (R = 0.998), respectively. The degradation rate of the BTB cross-linked PTMC_256_-0.1 was slightly lower than that of the BCP cross-linked ones. It was attributed to the difference in the gel percentages of the two groups as shown in Table 1. Similar to the results stated above, higher gel percentage and *T*_g_ induced reduction in the degradation rate. Here, we concluded that the BTB was more suitable to be utilized widely to fabricate the strong elastomeric polymer network with low degradation rate for biomedical applications.

The results of the degradation rate of the PTMC-Ns stated above strongly demonstrated that the chemical cross-linking was an efficient strategy to reduce the degradation rate of PTMC via the manipulation of the cross-linker amount, initial molecular weight as well as the cross-linker type, allowing their extensive use in the long-term biomedical applications.

### 3.3. SEM Observation

**Figure 5:**
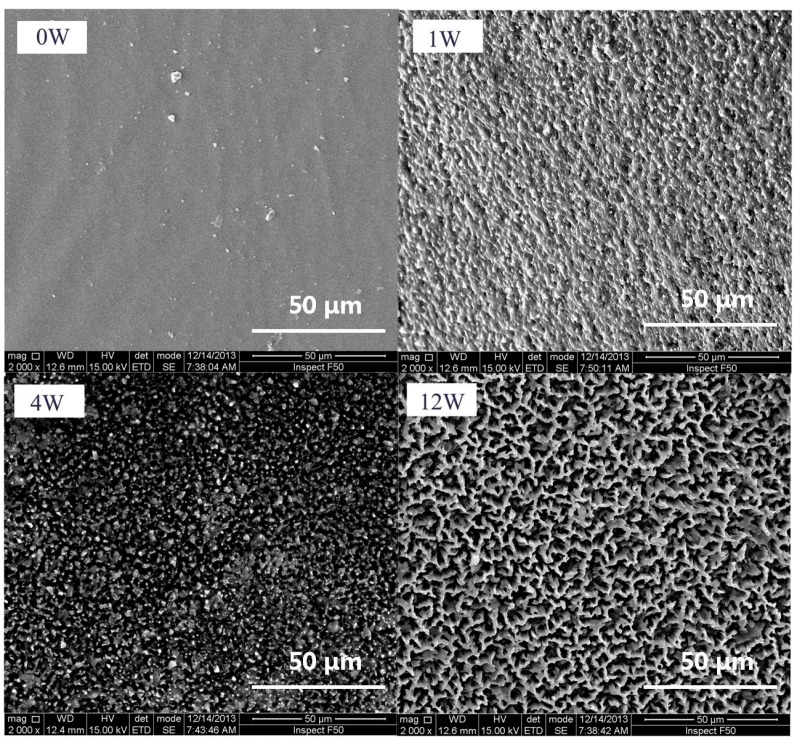
Scanning electron microscope (SEM) micrographs of PTMC_40_-0.1 before and after 1, 4 and 12 weeks’ enzymatic degradation.

The SEM measurements were performed to monitor the degradation of the resulting PTMC-Ns. Figure 5 shows the surface morphology of PTMC_329_-0.1 before and after degradation. The original samples of PTMC_329_-0.1 exhibited a smooth surface before enzymatic degradation. However, the surface became rough after 1 week of degradation, and a highly hollow structure was detected after four weeks, the size and deepness of the hollows observed on the surface increased with the incubation time, as illustrated in Figure 5. The results were similar to the findings reported in our previous works [30].

**Figure 6:**
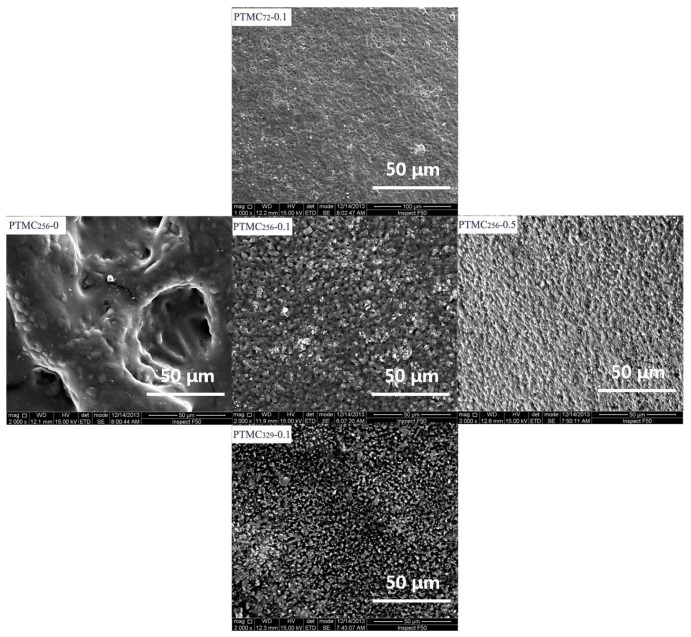
SEM micrographs of the PTMC-Ns surface with different cross-linker amount (Rank) and different initial molecular weight (Column) after four weeks degradation in lipase solution.

Figure 6 shows the surface morphology of the PTMC-Ns with different cross-linker amount and initial molecular weight after four weeks degradation in 37 °C lipase solutions. Obviously, the surface morphology varied with the formulation of the resulting PTMC-Ns. After four weeks of degradation, pits with greater size and deepness were observed on the surface of PTMC_256_-0, indicating that the surfaces had been extensively eroded. The pits on the surface of PTMC_256_-0.1 and PTMC_256_-0.5 became much shallower as indicated by the smaller roughness. That indicates the reduction in the erosion extent of the resulting networks with increasing cross-linker amount is very clear. Similar results were observed as the initial molecular weight decreased. The SEM results were coincidence with that of the mass loss.

### 3.4. Form-Stability and Degradation Mechanism

The form-stability of biodegradable polymers greatly influences their utility in the final biomedical devices, especially in those where deformation is not desired, e.g., the implant carriers for controlled drug release. Here, the form-stability of the resulting PTMC-Ns in vitro enzymatic degradation was determined via macroscopic observation.

**Figure 7:**
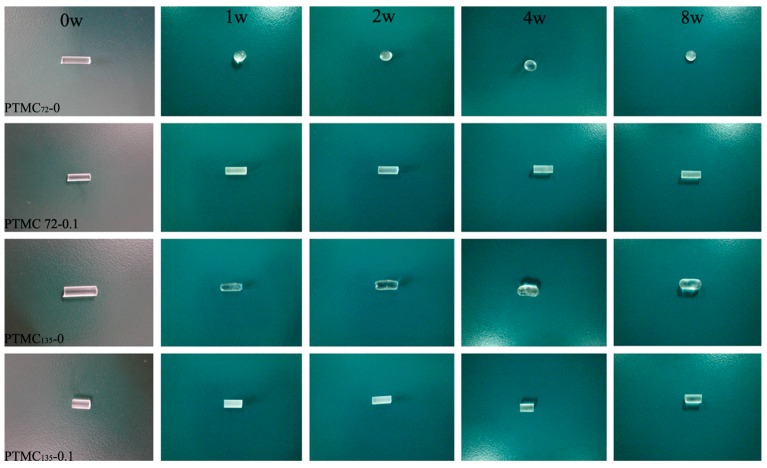
The macroscopic observation of the PTMC_72_-Ns and PTMC_135_-Ns at different times in 37 °C lipase solutions (0, 1, 2, 4, 8w represent before and after 1, 2, 4 and 8 weeks’ enzymatic degradation respectively).

Figure 7 shows that the uncross-linked low-molecular-weight PTMC rods (PTMC_72_-0 and PTMC_135_-0) splayed poor form-stability, the shape of the polymer rods was not cylindrical anymore, especially for the PTMC_72_-0 that underwent the worst deformation, and began changing its shape from cylinder to a highly sphere at week 1. Obviously, the corresponding cross-linked PTMC-Ns presented much better form-stability than the uncross-linked ones. As shown in Figure 7, the resulting networks PTMC_72_-0.1 and PTMC_135_-0.1 retained their initial appearance and no deformation was observed during the degradation process. Therefore, we deduced that cross-linking plays an important role in enhancing the form-stability of the PTMC-based polymers. In our previous works [21], we found that the uncross-linked high-molecular-weight PTMC (PTMC_256_-0 and PTMC_329_-0) could sustain their forms during the in vitro enzymatic degradation process, indicating their good form-stability. In this study, no difference was determined in the form-stability between the uncross-linked and cross-linked PTMC-Ns with high initial molecular weight (the figures were not displayed). The results illustrated that the cross-linking influenced the form-stability of the low-molecular-weight PTMC more significantly.

**Figure 8:**
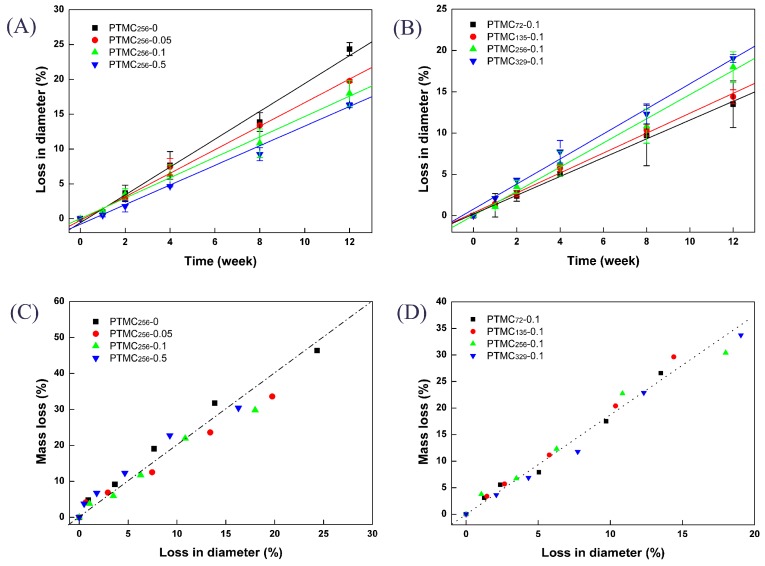
The loss in diameter (**A**,**B**) and the relationship between the mass loss and loss in diameter (**C**,**D**) of PTMC-Ns with different BTB amount (**A**,**C**) and different initial molecular weight (**B**,**D**) during degradation in 37 °C lipase solutions.

As seen in Figure 7, although the shape of PTMC-Ns was still rod-like independent of their initial molecular weight or the cross-linker amount, the diameter decreased upon enzymatic degradation. Figure 8A,B clearly shows the loss in diameter of the PTMC-Ns as a function of the degradation time. Apparently, the loss in diameter was dependent on the cross-linker amount and the initial molecular weight of PTMC-Ns. The higher the BTB amount, the lower the loss in diameter (Figure 8A), and the lower the initial molecular weight, the lower the loss in diameter (Figure 8B). The results were in a good agreement with that of the mass loss and degradation rates of PTMC-Ns.

The linear relationship between mass loss and loss in diameter, shown in Figure 8C,D, implied that the decrease in mass occurred simultaneously with a decrease in diameter, indicating that the in vitro enzymatic degradation of PTMC-Ns occurred via surface erosion mechanism. Similar results were observed for the BCP cross-linked PTMC_256_-0.1.

As the surface erosion mechanism worked on the enzymatic degradation of PTMC-Ns, the effective surface area of the degraded samples crucially influenced the degradation rate. Due to the poor form-stability, the effective surface area of the low-molecular-weight PTMC rods (PTMC_72_-0 and PTMC_135_-0) became smaller as they presented an appearance of oblate or sphere (Figure 7). Consequently, the degradation rate decreased with decreasing the surface area. The degradation rate of PTMC_72_-0 and PTMC_135_-0 calculated based on the increase of the mass loss was 0.94 and 1.46 %/w [21], respectively, much lower than that of the corresponding cross-linked ones. The results once again confirmed that the form-stability of biodegradable implants was an important property that should be paid high attention to, because it not only greatly influenced the final utility of the biomedical devices, but also played a significant role in the degradation behavior of the implants.

### 3.5. The Role of Lipase on Degradation of PTMC-Ns

**Figure 9:**
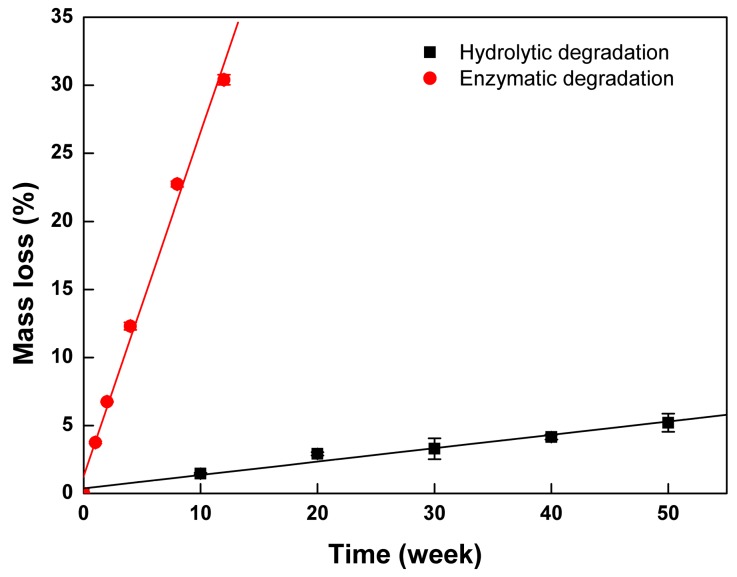
The degradation behavior of the PTMC_256_-0.1 with and without the lipase.

To elucidate the role of lipase in the degradation behavior of PTMC-Ns, the control experiment (hydrolytic degradation) without enzyme was also performed. The hydrolytic degradation of PTMC_256_-0.1 was investigated in pH 7.4 PBS. Figure 9 shows the mass loss of the PTMC_256_-0.1 degraded in the absence of lipase as a function of the degradation time, in comparison with that of the enzymolysis case.

It is clearly seen that the PTMC_256_-0.1 lost its mass much faster with lipase than without lipase. After 12 weeks, the mass loss of PTMC_256_-0.1 incubated in lipase was 30.4 ± 0.37%, whereas it only lost 5.20 ± 0.67% of its mass after 50 weeks of degradation without lipase. The degradation rate constant of PTMC_256_-0.1 in the presence and in the absence of the lipase were 2.52 and 0.098 %/w, respectively. The former was more than 25 times higher than the latter. The rapid surface erosion of PTMC-Ns in enzymatic conditions as well as stable under hydrolytic conditions suggested that enzymes played an important role in the degradation process. It was attributed to the fact that the lipase could act as a surfactant to interact with the degradation products of PTMC-Ns and accelerate them dispersing into the surrounding media. We concluded that the lipase could accelerate the degradation of PTMC-Ns by enhancing the loss of the degradation products, as observed in the in vitro degradation of the PTMC in our separate studies [21].

### 3.6. pH

**Figure 10:**
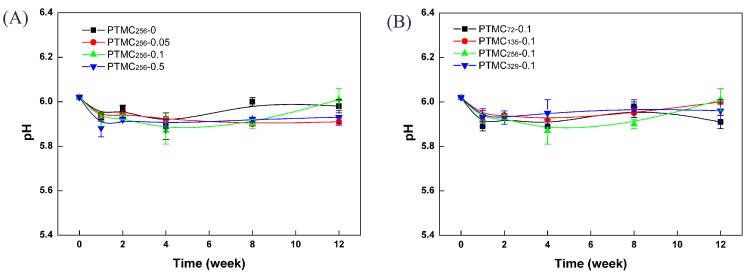
The pH profile of lipase solutions after enzymatic degradation of PTMC-Ns with: different cross-linker amount (**A**); and different initial molecular weight (**B**).

It has been reported that the acidic degradation compounds are not produced upon the degradation of PTMC [1,2,21]. As far as PTMC-Ns are concerned, the degradation products should be non-acidic because of the polycarbonate structure of PTMC-Ns. There are fair evidences to support this view from the changes in pH of the degradation media. During erosion studies, the enzyme solutions remained clear indicating that degradation products were water soluble. In Figure 10, the pH of the lipase solution was monitored with the degradation time. As expected, no acidification was seen when the networks degradation occurred. The pH of lipase was almost unchanged was close to the initial value of 6.02, strongly demonstrating that PTMC-Ns degraded without acidic products. Importantly, the property of releasing no acidic products was independent of the cross-linking amount and the initial molecular weight. The same result was observed for the BCP cross-linked PTMC-Ns. It is very important to eliminate the inflammatory reaction evoked by the acidic degradation products as observed in the cases of PLA, PGA and their copolymers [5,6,7]. In addition, no accelerated degradation induced by the autocatalysis [38] would take place during the degradation of PTMC-Ns, allowing them to be applied more widely in the biomedical fields.

## 4. Conclusions

The in vitro enzymatic degradation behavior of PTMC-Ns was quantitatively studied. The results illustrated that the degradation of PTMC-Ns was dependent on the initial molecular weight. Higher mass loss and subsequent higher degradation rate were detected for PTMC-Ns with higher initial molecular weight at a given cross-linker amount. The degradation rate of PTMC-Ns could also be tailored by the adjustment of cross-linker amount: the higher the cross-linker amount, the slower the degradation rate. Furthermore, the cross-linker type was also a valid means to tune the degradation rate of PTMC-Ns. The degradation rate of PTMC-Ns was slower than that of the linear ones, indicating the enhanced resistance to degradation of PTMC via cross-linking.

PTMC-Ns maintained better form-stability versus the non-crosslinked ones during the enzymatic degradation, indicating that cross-linking was an effective strategy to enhance the form-stability of PTMC. With the comparison of an in vitro hydrolysis case, the much faster degradation rate in lipase was attributed to the essential role of enzymes in the erosion of PTMC-Ns. We concluded that the lipase acted as a surfactant to promote the dissolution of the degradation products into the surrounding media and thus accelerate the degradation via surface erosion mechanism.

As stated above, PTMC-Ns with enhanced form-stability and lowered degradation rate given by cross-linking are the promising candidates for potential clinical application in subcutaneous implants. The degradation behavior with no acidic degradation products would donate well biocompatibility to PTMC-Ns. Future studies will focus on the in vivo degradation behaviors of and the tissue response to PTMC-Ns.

## Figures and Tables

**Table 1:** Properties of the resulting poly(trimethylene carbonate) networks (PTMC-Ns) (PTMC*x*-N*y*) fabricated in different cross-linking conditions (*x*: initial molecular weight; *y*: cross-linker amount), BCP: bis(ε-caprolactone).

PTMC-Ns	Initial Molecular Weight (kg/mol)	Cross-Linker Amount (mol %)	Cross-Linker Type	Gel Percentage (%)	Swelling Degree (%)	*T*_g_^a^ (°C)	*T*_d_^b^ (°C)	E ^c^ (MPa)	σm ^d^ (MPa)	εm ^e^ (%)
PTMC_256_-0	256	0	BTB	0	-	−14.3	258.9	3.1 ± 0.08	0.9 ± 0.1	-
PTMC_256_-0.05	256	0.05	BTB	80.9 ± 3.2	3943 ± 103	−13.4	282.2	9.5 ± 0.5	5.1 ± 0.06	420 ± 170
PTMC_256_-0.1	256	0.1	BTB	91.6 ± 2.4	1453 ± 52	−13.2	288.7	10.9 ± 0.8	6.2 ± 0.5	295 ± 72
PTMC_256_-0.5	256	0.5	BTB	97.9 ± 1.1	498 ± 16	−12.6	290.5	11.4 ± 1.0	7.2 ± 1.9	137 ± 12
PTMC_72_-0.1	72	0.1	BTB	39.9 ± 2.0	9916 ± 270	−13.7	267.0	6.3 ± 0.5	2.0 ± 0.07	507 ± 38
PTMC_135_-0.1	135	0.1	BTB	82.7 ± 0.5	2086 ± 11	−13.4	279.1	7.3 ± 0.4	4.0 ± 0.6	303 ± 49
PTMC_329_-0.1	329	0.1	BTB	98.0 ± 1.3	567 ± 25	−12.7	290.7	12.2 ± 0.3	8.0 ± 1.0	228 ± 32
PTMC_256_-0.1	256	0.1	BCP	89.7 ± 1.7	2203 ± 134	−13.5	283.8	9.7 ± 0.3	5.4 ± 0.6	-

^a^ *T*_g_ is the glass transition temperature from the second heating scan; ^b^ ***T*_d_** is the onset temperature of thermal degradation; ^c^ E is the Young’s modulus; ^d^ σm is the tensile stress; ^e^ εm is the tensile strain.

**Table 2:** The enzymatic degradation rate of BTB cross-linked PTMC-Ns with different cross-linker amount determined from the increase of mass loss.

PTMC-Ns	Rate Constant k
	%/w
PTMC_256_-0	3.8
PTMC_256_-0.05	2.8
PTMC_256_-0.1	2.5
PTMC_256_-0.5	2.5

**Table 3:** The enzymatic degradation rate of BTB cross-linked PTMC-Ns with different initial molecular weight determined from the increase of mass loss.

PTMC-Ns	Rate Constant k
	%/w
PTMC_72_-0.1	2.2
PTMC_135_-0.1	2.4
PTMC_256_-0.1	2.5
PTMC_329_-0.1	2.8

## References

[1] Zhu K.J., Hendren R.W., Jensen K., Pitt C.G. (1991). Synthesis, properties, and biodegradation of poly(1,3-trimethylene carbonate). Macromolecules.

[2] Zhang Z., Kuijer R., Bulstra S.K., Grijpma D.W., Feijen J. (2006). The in vivo and in vitro degradation behavior of poly(trimethylene carbonate). Biomaterials.

[3] Pêgo A.P., Van Luyn M.J., Brouwer L.A., van Wachem P.B., Poot A.A., Grijpma D.W., Feijen J. (2003). In vivo behavior of poly(1,3-trimethylene carbonate) and copolymers of 1,3-trimethylene carbonate with d,l-lactide or epsilon-caprolactone: Degradation and tissue response. J. Biomed. Mater. Res. Part A.

[4] Albertsson A.C., Eklund M. (1995). Influence of molecular structure on the degradation mechanism of degradable polymers: In vitro degradation of poly(trimethylene carbonate), poly(trimethylene carbonate-*co*-caprolactone), and poly(adipic anhydride). J. Appl. Polym. Sci..

[5] Athanasiou K.A., Niederauer G.G., Agrawal C.M. (1996). Sterilization, toxicity, biocompatibility and clinical applications of polylactic acid/polyglycolic acid copolymers. Biomaterials.

[6] Sachlos E., Czernuszka J.T. (2003). Making tissue engineering scaffolds work. Review: The application of solid freeform fabrication technology to the production of tissue engineering scaffolds. Eur. Cells Mater..

[7] Karp J.M., Shoichet M.S., Davies J.E. (2003). Bone formation on two-dimensional poly(dl-lactide-*co*-glycolide) (plga) films and three-dimensional plga tissue engineering scaffolds in vitro. J. Biomed. Mater. Res. Part A.

[8] Zhang Z., Foks M.A., Grijpma D.W., Feijen J. (2005). Ptmc and mpeg-ptmc microparticles for hydrophilic drug delivery. J. Control. Release.

[9] Zhang Y., Zhuo R.X. (2005). Synthesis and drug release behavior of poly (trimethylene carbonate)-poly (ethylene glycol)-poly (trimethylene carbonate) nanoparticles. Biomaterials.

[10] Gu F., Younes H.M., El-Kadi A.O., Neufeld R.J., Amsden B.G. (2005). Sustained interferon-gamma delivery from a photocrosslinked biodegradable elastomer. J. Control. Release.

[11] Yoshii T., Hafeman A.E., Nyman J.S., Esparza J.M., Shinomiya K., Spengler D.M., Mundy G.R., Gutierrez G.E., Guelcher S.A. (2010). A sustained release of lovastatin from biodegradable, elastomeric polyurethane scaffolds for enhanced bone regeneration. Tissue Eng. Part A.

[12] Guan J., Stankus J.J., Wagner W.R. (2007). Biodegradable elastomeric scaffolds with basic fibroblast growth factor release. J. Control. Release.

[13] Pêgo A.P., Poot A.A., Grijpma D.W., Feijen J. (2001). Copolymers of trimethylene carbonate and ε-caprolactone for porous nerve guides: Synthesis and properties. J. Biomater. Sci. Polym. Ed..

[14] Schappacher M., Fabre T., Mingotaud A.F., Soum A. (2001). Study of a (trimethylenecarbonate-*co*-ε-caprolactone) polymer—Part 1: Preparation of a new nerve guide through controlled random copolymerization using rare earth catalysts. Biomaterials.

[15] Fabre T., Schappacher M., Bareille R., Dupuy B., Soum A., Bertrandbarat J., Baquey C. (2001). Study of a (trimethylenecarbonate-*co*-epsilon-caprolactone) polymer—Part 2: In vitro cytocompatibility analysis and in vivo ed1 cell response of a new nerve guide. Biomaterials.

[16] Song Y., Wennink J.W., Kamphuis M.M., Vermes I., Poot A.A., Feijen J., Grijpma D.W. (2010). Effective seeding of smooth muscle cells into tubular poly(trimethylene carbonate) scaffolds for vascular tissue engineering. J. Biomed. Mater. Res. A.

[17] Bat E., Kothman B.H., Higuera G.A., van Blitterswijk C.A., Feijen J., Grijpma D.W. (2010). Ultraviolet light crosslinking of poly(trimethylene carbonate) for elastomeric tissue engineering scaffolds. Biomaterials.

[18] Song Y., Wennink J.W., Kamphuis M.M., Sterk L.M., Vermes I., Poot A.A., Feijen J., Grijpma D.W. (2011). Dynamic culturing of smooth muscle cells in tubular poly(trimethylene carbonate) scaffolds for vascular tissue engineering. Tissue Eng. Part A.

[19] Papenburg B.J., Schuller-Ravoo S., Bolhuis-Versteeg L.A., Hartsuiker L., Grijpma D.W., Feijen J., Wessling M., Stamatialis D. (2009). Designing porosity and topography of poly(1,3-trimethylene carbonate) scaffolds. Acta Biomater..

[20] Gui L., Zhao L., Spencer R.W., Burghouwt A., Taylor M.S., Shalaby S.W., Niklason L.E. (2011). Development of novel biodegradable polymer scaffolds for vascular tissue engineering. Tissue Eng. Part A.

[21] Yang L., Li J., Zhang W., Jin Y., Zhang J., Liu Y., Yi D., Li M., Guo J., Gu Z. (2015). The degradation of poly(trimethylene carbonate) implants: The role of molecular weight and enzymes. Polym. Degrad. Stab..

[22] Helminen A., Korhonen H., Seppälä J.V. (2001). Biodegradable crosslinked polymers based on triethoxysilane terminated polylactide oligomers. Polymer.

[23] Liu Q., Jiang L., Shi R., Zhang L. (2012). Synthesis, preparation, in vitro degradation, and application of novel degradable bioelastomers—A review. Prog. Polym. Sci..

[24] Yang L.-Q., He B., Meng S., Zhang J.-Z., Li M., Guo J., Guan Y.-M., Li J.-X., Gu Z.-W. (2013). Biodegradable cross-linked poly(trimethylene carbonate) networks for implant applications: Synthesis and properties. Polymer.

[25] Yang L., Li J., Jin Y., Zhang J., Li M., Gu Z. (2014). Highly efficient cross-linking of poly(trimethylene carbonate) via bis(trimethylene carbonate) or bis(ε-caprolactone). Polymer.

[26] Pêgo A.P., Grijpma D.W., Feijen J. (2003). Enhanced mechanical properties of 1,3-trimethylene carbonate polymers and networks. Polymer.

[27] Bat E., Plantinga J.A., Harmsen M.C., van Luyn M.J., Zhang Z., Grijpma D.W., Feijen J. (2008). Trimethylene carbonate and epsilon-caprolactone based (co) polymer networks: Mechanical properties and enzymatic degradation. Biomacromolecules.

[28] Bat E., Plantinga J.A., Harmsen M.C., van Luyn M.J., Grijpma D.W., Feijen J. (2010). In vivo behavior of trimethylene carbonate and ε-caprolactone-based (co) polymer networks: Degradation and tissue response. J. Biomed. Mater. Res. Part A.

[29] Bat E., van Kooten T.G., Harmsen M.C., Plantinga J.A., van Luyn M.J., Feijen J., Grijpma D.W. (2013). Physical Properties and Erosion Behavior of Poly (trimethylene carbonate-co-ε-caprolactone) Networks. Macromol. Biosci..

[30] Yang L., Li J., Li M., Gu Z. (2016). The in vitro and in vivo degradation of cross-linked poly(trimethylene carbonate)-based networks. Polymers.

[31] Yang L., Li J., Meng S., Jin Y., Zhang J., Li M., Guo J., Gu Z. (2014). The in vitro and in vivo degradation behavior of poly (trimethylene carbonate-*co*-ε-caprolactone) implants. Polymer.

[32] Matsumura S., Harai S., Toshima K. (2001). Lipase-catalyzed transformation of poly(trimethylene carbonate) into cyclic monomer, trimethylene carbonate: A new strategy for sustainable polymer recycling using an enzyme. Macromol. Rapid Commun..

[33] Jeon O., Song S.J., Lee K.J., Park M.H., Lee S.H., Hahn S.K., Kim S., Kim B.S. (2007). Mechanical properties and degradation behaviors of hyaluronic acid hydrogels cross-linked at various cross-linking densities. Carbohydr. Polym..

[34] Marten E., Müller R.-J., Deckwer W.-D. (2003). Studies on the enzymatic hydrolysis of polyesters I. Low molecular mass model esters and aliphatic polyesters. Polym. Degrad. Stab..

[35] Li S.M., Garreau H., Vert M. (1990). Structure-property relationships in the case of the degradation of massive aliphatic poly-(α-hydroxy acids) in aqueous media. J. Mater. Sci. Mater. Med..

[36] Chen B., Yin C., Cheng Y., Li W., Cao Z.-A., Tan T. (2012). Using silk woven fabric as support for lipase immobilization: The effect of surface hydrophilicity/hydrophobicity on enzymatic activity and stability. Biomass Bioenergy.

[37] Chen G.J., Kuo C.H., Chen C.I., Yu C.C., Shieh C.J., Liu Y.C. (2012). Effect of membranes with various hydrophobic/hydrophilic properties on lipase immobilized activity and stability. J. Biosci. Bioeng..

[38] Bergsma J.E., de Bruijn W.C., Rozema F.R., Bos R.R., Boering G. (1995). Late degradation tissue response to poly(l-lactide) bone plates and screws. Biomaterials.

